# Cladosporium sphaerospermum as a Rare Cause of Pneumonia

**DOI:** 10.7759/cureus.26256

**Published:** 2022-06-23

**Authors:** Diana M Villanueva, Bhuvaneshwari Venkatesan, Nilka Figueroa

**Affiliations:** 1 Department of Internal Medicine, Coney Island Hospital, Brooklyn, USA; 2 Department of Infectious Diseases, Coney Island Hospital, Brooklyn, USA

**Keywords:** hydropneumothorax, squamous cell carcinoma (scc), rare lung diseases, large pleural effusion, azole antifungal, acute hypoxemic respiratory failure, fungal lung infection, atypical pneumonia, fungal pneumonia, cladosporium sphaerospermum

## Abstract

*Cladosporium sphaerospermum* isa radiotrophic dematiaceous fungus that can rarely cause disease in humans such as infections of the skin, eye, upper airways, and brain. To the best of our knowledge, we present the first reported case of *Cladosporium sphaerospermum*-induced invasive lung infection. This case presents a 51-year-old male with a medical history significant for heavy smoking and severe alcohol abuse who was admitted for acute hypoxic respiratory failure secondary to a large exudative right pleural effusion compounded by hydropneumothorax. Despite an initial positive clinical response, appropriate medical treatment, and eradication of the infection, which was confirmed by repeat negative culture studies, the patient had a complicated hospital course. It is suspected that the patient’s medical history played a role in the acquisition of the *Cladosporium sphaerospermum* infection as smoking and alcohol use are known risk factors for aspiration of pathogens into the pulmonary tract. We believe it is important to bring to attention this less known organism as a potential differential diagnosis for a complicated lung infection.

## Introduction

*Cladosporium* species are typically facultative opportunistic human pathogens known to cause allergic reactions and occasionally cause pathologic disease in humans. Infections of the skin, eye, upper airways, and brain have been reported [1-5]. To date, only five cases of *Cladosporium*-induced pulmonary infections have been reported [6-10]. Four of the reported cases involve the subspecies *Cladosporium cladosporioides* [6-9]. We present the second reported case of *Cladosporium sphaerospermum*-associated pulmonary pathology to increase awareness of this pathogen as an important differential for the chest pathophysiologic process.

## Case presentation

**Figure 1:**
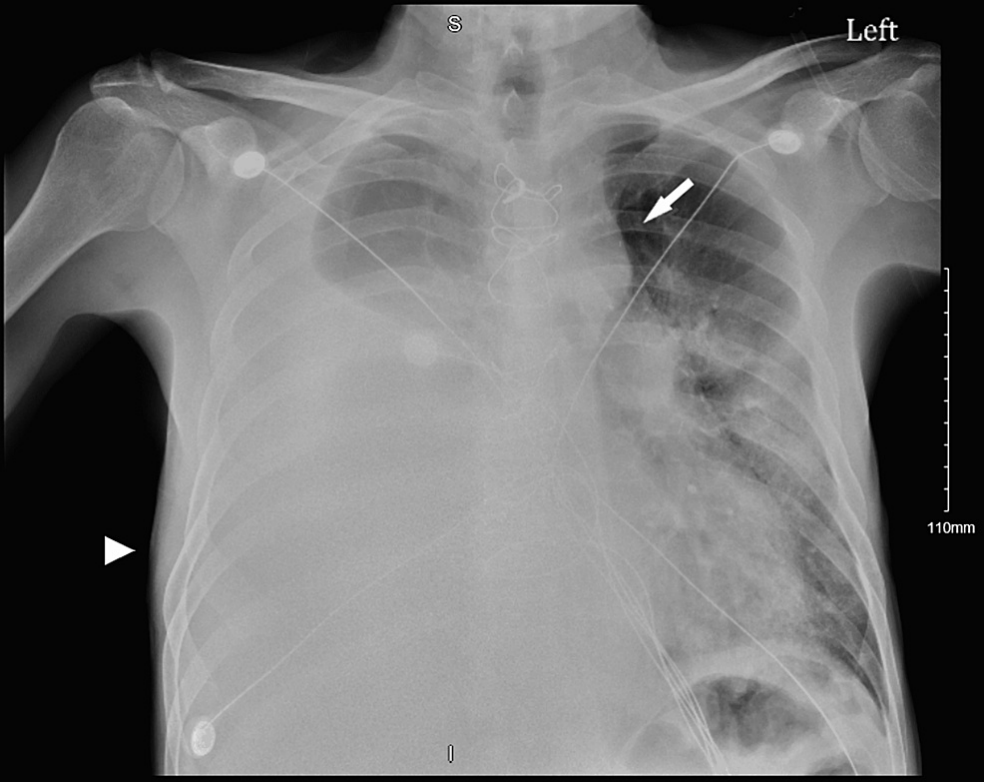
Chest radiograph. Chest radiograph depicting a large right pleural effusion causing opacification of the right hemithorax (arrowhead), with an associated shift of the heart and mediastinal structures into the left hemithorax (arrow).

A 51-year-old male with a medical history of hypertension, coronary artery disease, coronary artery bypass grafting (CABG), heavy smoking, severe alcohol use disorder with associated gastritis, esophagitis, and non-bleeding Mallory-Weiss tears presented to the emergency room inebriated with a complaint of difficulty breathing. In the emergency room, the patient was normotensive (blood pressure 118/66 mmHg), tachycardic (heart rate of 112 beats per minute), tachypneic (respiratory rate of 26 breaths per minute), afebrile (temperature of 36.9°C), and desaturating on room air (oxygen saturation of 65%), requiring 15 L of supplemental oxygen delivered via a non-rebreather mask. Physical examination was significant for decreased breath sounds in the right hemithorax and bilateral submandibular and cervical lymphadenopathy. The patient was admitted to the medical intensive care unit for the management of acute hypoxic respiratory failure due to pneumonia of an unknown organism, for which he was started on broad-spectrum antibiotics with cefepime, vancomycin, and azithromycin. A chest radiograph revealed a large right pleural effusion (Figure 1).

**Figure 2:**
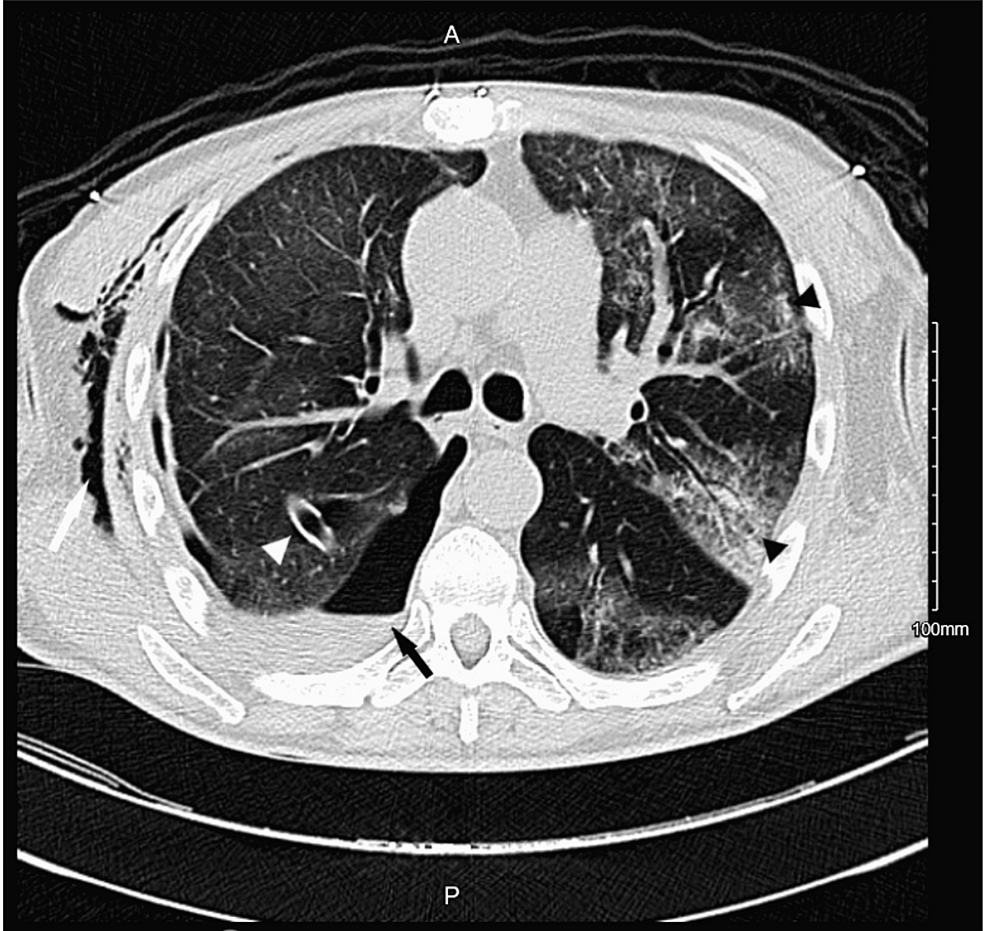
Computed tomography of the chest. Computed tomography of the chest depicting a right hydropneumothorax (black arrow) with associated right lower lobe atelectasis, diffuse bilateral ground-glass opacities suggestive of a pneumonic process (black arrowheads), right subcutaneous emphysema (white arrow), and right-sided chest tube coursing along the right major fissure (white arrowhead).

A chest tube was placed to drain the effusion, which yielded 2.5 L of serosanguinous fluid. Computed tomography (CT) of the chest unveiled a right hydropneumothorax with right lower lobe consolidation, as well as diffuse bilateral ground-glass opacities (Figure 2).

The pleural fluid was determined to be exudative in nature as per fluid analysis, and the pleural culture was positive for *Cladosporium sphaerospermum*. Lymph node biopsy of a palpable posterior cervical nodule was positive for squamous cell carcinoma of the base of the tongue. The Oncology Department was consulted, with recommendations for immediate initiation of treatment.

The patient was noted to clinically improve with the empiric antibiotic treatment regimen started on admission even though the infection was later determined to be fungal in nature. The Infectious Diseases Department was consulted for recommendations regarding medical treatment and duration of therapy. All antibiotics were discontinued, and the patient was treated with 200 mg of voriconazole every 12 hours for 14 days in preparation for upcoming chemotherapy. Repeat fungal cultures from the pleural fluid were negative. However, the patient’s hospital course was complicated by worsening hydropneumothorax requiring video-assisted thoracic surgery (VATS) decortication, which aided in the resolution of the complicated pleural effusion.

## Discussion

*Cladosporium sphaerospermum* is a radiotrophic dematiaceous fungus that is typically found in decaying leaves and branches of citrus trees. *Cladosporium sphaerospermum* is an allergen and, in rare cases, a cause of human disease which has been reported to cause infections of the skin [1-3], eye, sinus [4], and brain [5]. To the best of our knowledge, this will be the second reported case of *Cladosporium sphaerospermum*-induced pulmonary infection. Hence, we report the case of a 51-year-old male with a medical history significant for smoking and alcohol use disorder who was admitted for acute hypoxic respiratory failure secondary to a large exudative pleural effusion with pleural cultures positive for *Cladosporium sphaerospermum*.

A review of the literature yielded five pulmonary-related *Cladosporium* species infections, but only one of the case reports mentions *Cladosporium sphaerospermum* as the causative organism. The first reported case dates back to 1975 and describes a right upper lobe fungal ball [6]. The second describes the case of a 43-year-old woman who presented with atelectasis of the left lung caused by obstruction of the left main bronchus by necrotic mucoid lesion [7]. The third is the case of an immunocompetent patient with an intrabronchial lesion that was treated with an intrabronchial infusion of amphotericin B, prednisolone, and itraconazole [8]. The fourth is the case of a 59-year-old male outpatient who presented with hemoptysis and was found to have hemorrhagic pneumonia [9]. The only other report describing *Cladosporium sphaerospermum *as a cause of pulmonary pathology is the case of *Cladosporium*-related hypersensitivity pneumonitis in household environments [10]. To the best of our knowledge, our case report is the first reported case of *Cladosporium sphaerospermum *resulting in a large pulmonary effusion complicated by the development of pneumothorax and the need for VATS decortication.

The extensive smoking history of the patient presented in this report, compounded with his higher risk for aspiration given his severe alcohol use disorder, was suspected to have been contributing factors in the acquisition of the pulmonary infection by *Cladosporium sphaerospermum.*

## Conclusions

*Cladosporium species* are facultative opportunistic human pathogens that typically cause infections of the skin, eye, upper airways, and brain; however, rarely, *Cladosporium *species such as *Cladosporium sphaerospermum* can infect other organ systems, such as the pulmonary system, leading to severe disease. The presented case is, to the best of our knowledge, the first reported case of *Cladosporium sphaerospermum*-induced invasive lung infection, leading to the development of complicated pneumonia. Despite an initial positive clinical response, appropriate medical treatment, and eradication of the infection which was confirmed by repeat negative culture studies, the patient experienced further complications requiring surgical intervention. A higher risk for aspiration of pathogens, explained by the patient’s significant smoking history and severe alcohol use disorder, is suspected to have played a role in the acquisition of *Cladosporium sphaerospermum*; however, further studies are needed to establish whether a correlation exists. Moreover, it is plausible that the patient’s active malignancy may have resulted in an immunocompromised state, rendering the patient prone to infections with atypical organisms. It is important to keep *Cladosporium *species in the differential diagnosis as it can lead to increased morbidity and serious hospital course complications.
